# Effects of Cardiac Hypertrophy, Diabetes, Aging, and Pregnancy on the Cardioprotective Effects of Postconditioning in Male and Female Rats

**DOI:** 10.1155/2019/3403959

**Published:** 2019-05-06

**Authors:** Fawzi Babiker, Aishah Al-Jarallah, Mariam Al-Awadi

**Affiliations:** ^1^Department of Physiology, Faculty of Medicine, Health Science Center, Kuwait University, Jabriya, Kuwait; ^2^Biochemistry, Faculty of Medicine, Health Science Center, Kuwait University, Jabriya, Kuwait

## Abstract

**Background:**

Aging, left ventricular hypertrophy (LVH), diabetes mellitus, and pregnancy are well-recognized risk factors that increase the prevalence of cardio-ischemic events and are linked to poor clinical recovery following acute myocardial infarction. The coexistence of these risk factors with ischemic heart disease (IHD) deteriorates disease prognosis and could potentially lead to fatal arrhythmias and heart failure. The objective of this study was to investigate the vulnerability of hearts with aging, LVH, diabetes, and pregnancy to ischemic insult and their response to pacing postconditioning- (PPC-) induced heart protection.

**Methods:**

Hearts isolated from aged, spontaneously hypertensive and diabetic male and female rats and hearts from pregnant female rats (*n*=8 per group) were subjected to coronary occlusion followed by reperfusion using a modified Langendorff system. Hemodynamics data were computed digitally, and cardiac damage was accessed by measurements of infarct size and cardiac enzyme release.

**Results:**

There were no significant differences in the vulnerability of all hearts to ischemic insult compared to their respective controls. PPC improved cardiac hemodynamics and reduced infarct size and cardiac enzyme release in hearts isolated from aged and spontaneously hypertensive female rats and female rats with hypertrophied hearts subjected to PPC (*P* < 0.001). Aged or hypertrophied male hearts were not protected by PPC maneuver. Moreover, the protective effects of PPC were lost in diabetic male and female hearts although retained in hearts from pregnant rats.

**Conclusions:**

We demonstrate that aging, LVH, diabetes mellitus, and pregnancy do not affect cardiac vulnerability to ischemic insult. Moreover, PPC mediates cardioprotection in a gender-specific manner in aged and spontaneously hypertensive rats. Diabetes mellitus provokes the protective effects of PPC on both genders equally. Finally, we demonstrate that PPC is a new cardioprotective maneuver in hearts from pregnant female rats.

## 1. Background

Ischemic heart disease (IHD) remains a global health concern, and acute myocardial infarction continues to be a leading cause of death [1]. Restriction of coronary blood supply leads to myocardial infarction which is the primary determinant of disease prognosis [1]. The infarct size and disease outcome can remarkably be improved by restoration of blood flow by reperfusion [2]. However, reperfusion itself can be devastating resulting in significant damage within the ischemia-affected area [2]. Several cardioprotective strategies have been developed to limit the destructive effects of ischemia/reperfusion (I/R) injury on the myocardium. These include, but are not limited to, the application of brief sublethal ischemic episodes at the beginning of reperfusion on-site or a distant vascular bed (ischemic postconditioning and remote ischemic postconditioning, respectively) [3, 4]. More recently, a modification of the remote postconditioning maneuver has been introduced which involves changing myocardial stretch pattern via remote electrical stimulation of the heart chambers (pacing postconditioning (PPC)) [5]. We have reported that PPC is an effective protective postconditioning maneuver against I/R injury in experimental animals [5–7]. We have further investigated the molecular mechanisms involved in PPC-induced cardiac protection [8–10]. The first clinical application of the PPC maneuver demonstrated significant decrease in infarct size in ST-segment elevation myocardial infarction (STEMI) patients [11]. This, however, was accompanied by arterial and ventricular fibrillation [11]. Additional clinical studies would, therefore, require further technical optimization and more comprehensive consideration of various clinical scenarios existing in MI patients that could potentially attenuate the cardioprotective properties of PPC or may cause undesirable side effects.

Aging-induced structural and functional changes in the cardiovascular system were linked to increased incidence of CVD in the elderly [12]. Aging is a common risk factor in MI patients and reduces patients' tolerance to ischemia and imposes a significant impact on disease prognosis [13, 14]. Ischemic postconditioning-induced cardiac protection was attenuated in senescent mice [15]. The efficiency of PPC-induced cardiac protection in aging remains to be established.

Increased susceptibility of hypertrophied heart to ischemia [16] and postischemic arrhythmias [17] were previously reported. Available evidence on the efficiency of ischemic postconditioning strategies in attenuating ischemic injury in the presence of LVH is contradictory, possibly due to use of different LVH models [18, 19]. LVH has been associated with increased mortality from MI [20]. Thus, the identification of effective maneuvers for the protection of hypertrophied hearts is essential.

Patients with type 1 and type 2 diabetes are prone to cardiovascular diseases [21], and IHD remains to be a significant source of morbidity and mortality in these patients. Increased risk of ischemic events and poor recovery after an acute MI event were demonstrated in diabetic patients [22]. Inconsistent data from experimental models were reported on the susceptibility of diabetic heart to ischemic injury [23, 24]. The available data on the efficacy of postconditioning strategies in protecting the diabetic heart from ischemic injury are equivocal [25, 26], possibly due to the use of different experimental models and conditioning protocols. We have previously reported that seven cycles of alternating left and right ventricular pacing for 30 seconds each to a minimum of 200 seconds did not protect diabetic rabbit hearts from I/R injury [25]. Nonetheless, the effect of PPC in protecting diabetic rodent hearts against ischemic injury has not been previously given consideration.

There is a notable increase in the incidence of IHD in pregnancy in the recent years, possibly due to the increasing maternal age, higher prevalence of cardiac risk factors, and change of some social habits like smoking [27]. Enhanced risk of IHD and deteriorated prognosis were reported in pregnancy [27]. Pregnancy causes significant changes in cardiac hemodynamics which could ultimately lead to IHD [28]. Also, increased cardiac workload and increased myocardial oxygen demand during pregnancy may cause severe IHD [29]. Although many treatment regimens were used in treating IHD during pregnancy, postconditioning data in animal models or clinical studies are lacking [6, 30].

In this study, we were set to investigate the efficacy of PPC maneuver in protection against I/R injury in male and female rats in the presence of clinically relevant scenarios including aging, LVH, diabetes mellitus, and pregnancy.

## 2. Materials and Methods

### 2.1. Materials

All materials were purchased from Sigma-Aldrich (St. Louis, Missouri) unless stated otherwise.

### 2.2. Animals and Procedures

**Figure 1:**
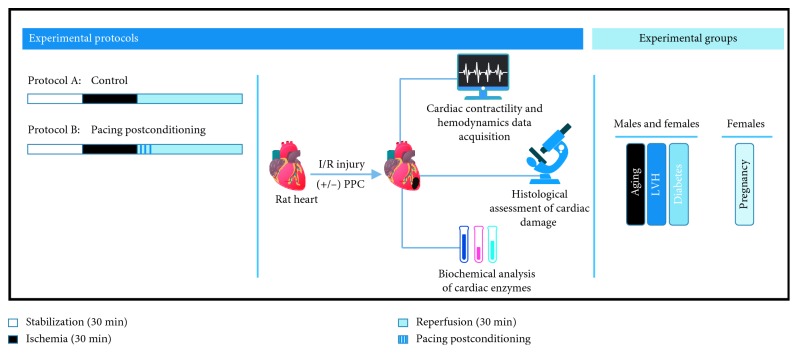
Illustration of the protocols and experimental groups used in the study. LVH, left ventricle hypertrophy.

Animal treatments and handling were performed according to the laboratory animal care guidelines of Kuwait University in accordance with International Guide for the Care and Use of Laboratory Animals (Eighth edition, 2011). In this study, we followed methods and procedures used in our previous studies [6, 25, 31]. Age-matched male and female Sprague–Dawley rats weighing between 210 and 370 g were used in this study. The rats were maintained at 22°C on a 12-hr light/dark cycle (7 am–7 pm), and water and food were provided ad libitum. A total of 128 rats were subdivided into 16 groups (*n*=8 per group) and were subjected to 4 different experimental protocols, as summarized in (Figure 1). All rats used in the study are 20 weeks old unless otherwise stated. To study the effects of aging and LVH, hearts from 20-month-old male and female SD rats and spontaneously hypertensive rats (SHR) were used, respectively. For experiments on diabetic hearts, diabetes was induced by a single intraperitoneal injection of 55 mg/kg body weight streptozotocin (STZ) as described previously [32]. Basal glucose levels were determined before the STZ injection and 48 hr after injection. Blood glucose concentration of 250 mg/dL was used as a cutoff value. Rats with blood glucose concentrations more than 250 mg/dL were declared diabetic while rats that did not meet this criterion were excluded from the study [33]. The animals' diabetic state was re-assessed six weeks later, just before sacrifice. For pregnancy studies, rats were mated overnight. The next day, vaginal smears were examined for the presence of sperms. Females with sperm-positive smear were identified as pregnant and designated day 0 of pregnancy as described previously [34]. These rats were weighed and individually housed in Plexiglas cages undisturbed until day 19 or 21 of gestation when they were sacrificed. 19 and 21 gestation days were selected based on the critical changes in the pregnancy progress in these animals [35].

Anesthesia was administered via an intraperitoneal injection of sodium pentobarbital (60 mg/kg). Heart cannulation and perfusion were performed as described previously [6]. Briefly, the isolated heart was retrogradely perfused with freshly prepared Krebs–Henseleit buffer. Oxygenation was performed using a mixture of CO_2_ (5%) and O_2_ (95%) at a temperature of 37.0 ± 0.5°C. Heart instrumentation included placing pacing electrodes on the right atrium (RA) appendage to maintain physiological heartbeats. Regional ischemia was induced by occluding the left coronary artery for 30 min. Under the basal control conditions, the preload was held constant at 6 mmHg. The perfusion pressure (PP) was held constant at 50 mmHg throughout the experimental procedure in all protocols. The PP was measured immediately downstream of the flow probe from a branch of the aortic cannula using a Statham pressure transducer (P23 Db). Constant PP was ensured electronically using the perfusion assembly (Module PPCM type 671, Hugo Sachs Elektronik, Harvard Apparatus GmbH, Germany), an effective system for accurate adjustment of PP between 5 mm Hg and 150 mm Hg with an accuracy of ±1 mmHg.

### 2.3. Study Protocol

All hearts were subjected to 30 min of ischemia produced by left coronary artery occlusion as described previously by Khalaf et al. [31]. Briefly, the left coronary artery was encircled by a snare approximately 0.5 cm below the atrioventricular (AV) groove, and a small rigid plastic tube was positioned between the heart and the snare to ensure complete occlusion of the coronary artery. Afterward, the hearts were reperfused for 30 min. Control hearts were subjected to I/R injury without any further treatment. The PPC involved three alternate RA and LV pacing episodes for 30 sec each. The on-and-off pacing cycles were selected based on our previous studies [10, 25]. The pacing electrode was fixed to the posterior basal LV wall and connected to a pacemaker set to the required pacing frequency. Simultaneous AV pacing (AV interval = 0 ms) was used for LV pacing to ensure complete ventricular activation in response to stimulation from the pacing electrode.

### 2.4. Evaluation of Heart Function

Left ventricular hemodynamics, contractility, and coronary-vascular dynamics were evaluated during stabilization, ischemia, and reperfusion periods. The LV dynamics were determined throughout the experiment by assessing the LV end-diastolic pressure (LVEDP), the maximum developed pressure (DPmax), and LV contractility (+dP/dt or −dP/dt). The coronary vascular dynamics were evaluated by assessing coronary flow (CF) and coronary vascular resistance (CVR). Cardiovascular functions were measured as previously described [6, 25, 31]. Briefly, a water-filled latex balloon was placed and secured in the LV cavity. The balloon was attached to a pressure transducer and a direct current bridge amplifier (DC-BA) equipped with a pressure module (DC-BA type 660, Hugo-Sachs Electronik, Germany) and interfaced to a personal computer for online monitoring of DPmax. The LVDP data were derived from online acquisition of LVEDP using the Max-Min module (Number MMM type 668, Hugo Sachs Elektronik, Harvard Apparatus GmbH, Germany), which converts the output from the DC-BA to DPmax by subtracting LVEDP.

CF was continuously measured using an electromagnetic flow probe attached to the inflow of the aortic cannula interfaced to a personal computer as described previously in Khalaf et al. [31]. The continuous monitoring of CF in ml/min was digitally monitored using the software developed by Hugo-Sachs (Hugo-Sachs Electronik, Germany) specifically for this purpose and was manually verified via the timed collection of the coronary effluent. The CVR and hemodynamic data were recorded every 10 sec using an online data acquisition program (Isoheart software V 1.524-S, Hugo-Sachs Electronik, Germany). At the end of each experiment, the heart was snap-frozen in liquid nitrogen and stored at −80°C for further analysis.

### 2.5. Evaluation of Cardiac Injury by Measurements of Infarct Size and Cardiac Enzymes Levels

The infarct size was determined by triphenyltetrazolium chloride (TTC) staining. Hearts were collected after 30 min of reperfusion and stored overnight at −20°C. The next day, each heart was sliced into 4-5 pieces from its apex to base along the long axis. The slices were then incubated for 15 min in 1% TTC solution in isotonic phosphate buffer (pH 7.4) at 37°C and fixed in 4% formaldehyde. Images were taken using Nikon camera. Red and pale unstained areas of every slice were indicated manually on the image using Leica ImageJ (Image J, Wayne Rasb and National Institute of Health, USA). The percentage infarct area was calculated relative to total LV area. Cardiomyocyte injury was evaluated by measuring creatine kinase (CK) and lactate dehydrogenase (LDH) release in the coronary effluent during the reperfusion period as previously described [36].

### 2.6. Data Analysis

Data were analyzed by two-way analysis of variance (ANOVA) followed by the least significant difference (LSD) post hoc analysis using SPSS software. Comparisons were performed between group means and the mean for their respective controls. The data were presented as the mean ± standard error of the mean and differences were considered statistically significant at *P* < 0.05.

## 3. Results

### 3.1. PPC Maneuver Provided Cardiac Protection in Aged Female but Not in Male Rats

**Figure 2:**
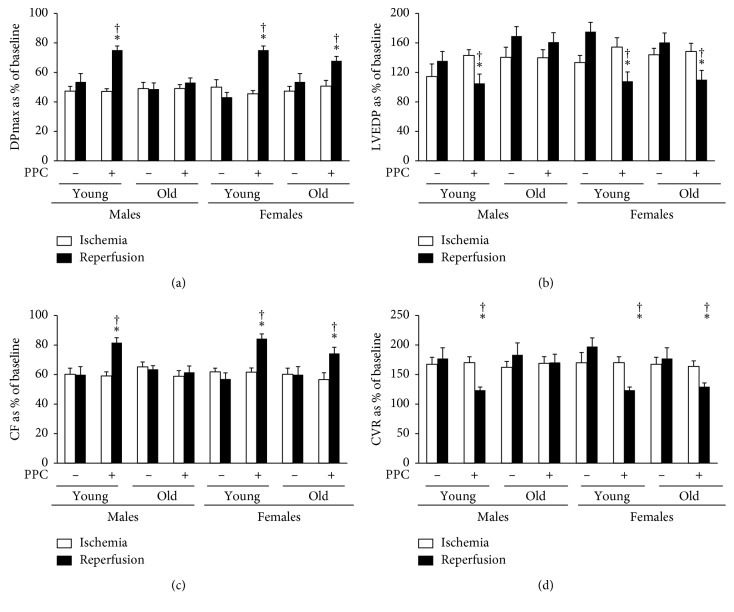
Effects of PPC on left ventricular and coronary vascular dynamics in hearts isolated from young and aging rats. PPC-mediated protection of the heart against I/R injury in hearts isolated from 20-month-old male and female rats (*n*=6 per group). Postischemic recovery from the dysfunction of the heart: (a) DPmax; (b) LVEDP; (c) CF; and (d) CVR. The data were computed at 30 min of reperfusion and are expressed as the means ± SEM. PPC, pacing postconditioning. ^*∗*^*P* < 0.01 compared to the respective control. ^†^*P* < 0.01 compared to the ischemic period.

**Figure 3:**
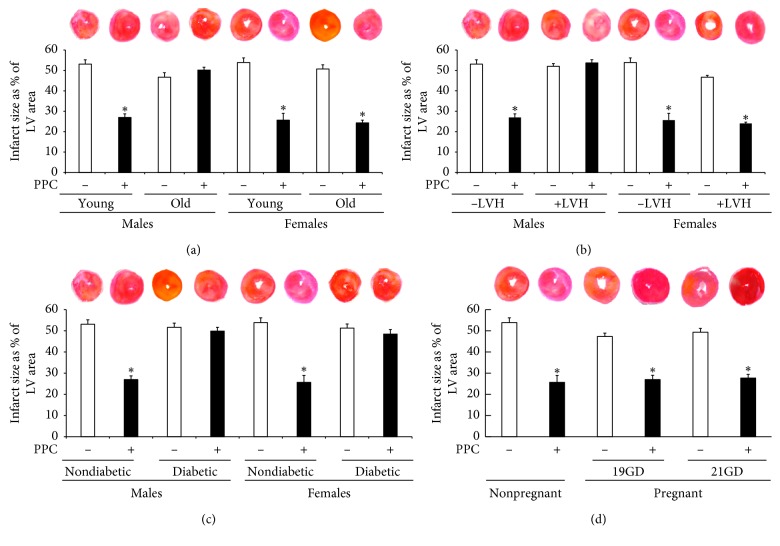
Histological assessment of ischemic injury. Infarct size presented area as a percentage of left ventricle area in the experimental models of aging (a), left ventricular hypertrophy (b), diabetes mellitus (c), and pregnancy (d). PPC, pacing postconditioning. ^*∗*^*P* < 0.01 compared to the respective control.

To address the role of age and gender, the effectiveness of PPC in protecting against I/R injury was evaluated in young (four months) and old (twenty months) male and female rats. There were no significant differences in the heart or body weights corrected to tibia length (mg/mm and g/mm, respectively) between young or old males and females. Heart functions were evaluated by the recovery of LV dynamics (DPmax and LVEDP), LV contractility (+dP/dt or −dP/dt), and coronary vascular dynamics (CF and CVR). Baseline changes in the LV dynamics and coronary vascular dynamics during baseline RA pacing were similar in young and aged male and female rats. Index Ischemia resulted in a significant (*P* < 0.001) deterioration in LV dynamics and coronary vascular dynamics compared to baseline (Figure 2). The vulnerability of hearts isolated from aged male and female rats to I/R injury was not significantly different from hearts isolated from younger male and female rats (Figure 2). The PPC intervention significantly (*P* < 0.05) improved LV dysfunction as evidenced by enhanced DPmax and reduced LVEDP in hearts isolated from aged female rats but not aged male rats (Figures 2(a) and 2(b)). In aged rats, PPC significantly improved coronary vascular dynamics, as indicated by enhanced CF and reduced CVR (*P* < 0.01) (Figures 2(c) and 2(d)), and improved myocardial contractility (*P* < 0.01) only in hearts isolated from females (Table 1). Consistent with the physiological data on cardiac functions, PPC significantly decreased the levels of cardiac enzymes (*P* < 0.01) and the myocardial infarct size (*P* < 0.01) in hearts from old female but not male rats (Figure 3(a) and Table 2). Taken together, the physiological, biochemical, and histological data indicate that PPC is an effective cardioprotective method in hearts isolated from young male and female rats and in hearts isolated from old female but not aged male rats.

### 3.2. PPC-Induced Cardiac Protection Is Revoked in Males with Left Ventricular Hypertrophy

**Figure 4:**
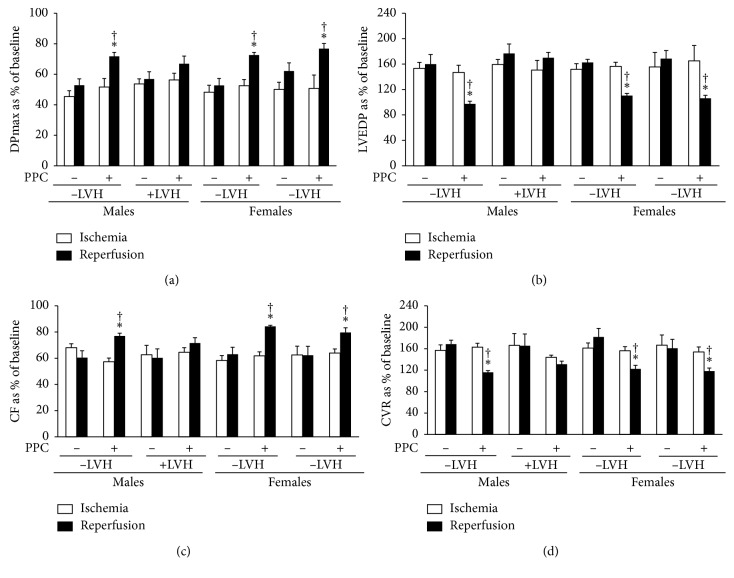
Effects of PPC on left ventricular and coronary vascular dynamics in hypertrophied and nonhypertrophied hearts. PPC-mediated protection in hearts with left ventricle hypertrophy (*n*=6 per group). Postischemic recovery from the dysfunction of the heart: (a) DPmax; (b) LVEDP; (c) CF; and (d) CVR. The data were computed at 30 min of reperfusion and are expressed as the means ± SEM. PPC, pacing postconditioning. ^*∗*^*P* < 0.01 compared to the respective control. ^†^*P* < 0.01 compared to the ischemic period.

In this set of experiments, we investigated the effect of LVH on the heart tolerance to ischemic injury and the effectiveness of PPC maneuver in its protection against I/R injury. Body weight corrected to tibia length was significantly (*P* < 0.05) higher in SHR males (289.73 ± 23.77) relative to the females (215.25 ± 2.73). Moreover, hearts of SHR males showed hypertrophy as indicated by a significant (*P* < 0.01) increase in heart weight corrected to tibia length (males, 0.39 ± 0.02; females, 0.34 ± 0.01). PPC maneuver was protective in nonhypertrophied hearts from males and females (Figures 3 and 4; Tables 1 and 2). Moreover, PPC did not induce a significant reduction in infarct size (Figure 2(b)) or cardiac enzyme release (Table 2). Interestingly however, PPC significantly improved left ventricular and coronary vascular dynamics (*P* < 0.001) (Figure 4 and Table 1), reduced infarct size (*P* < 0.001) (Figure 2(b)), and cardiac enzyme release (*P* < 0.01) (Table 2) in nonhypertrophied hearts from SHR females suggesting that the effectiveness of PPC as a cardioprotective maneuver is compromised in hypertrophied hearts.

### 3.3. Diabetes Mellitus Revokes the Cardioprotective Effects of PPC in Male and Female Rats

**Figure 5:**
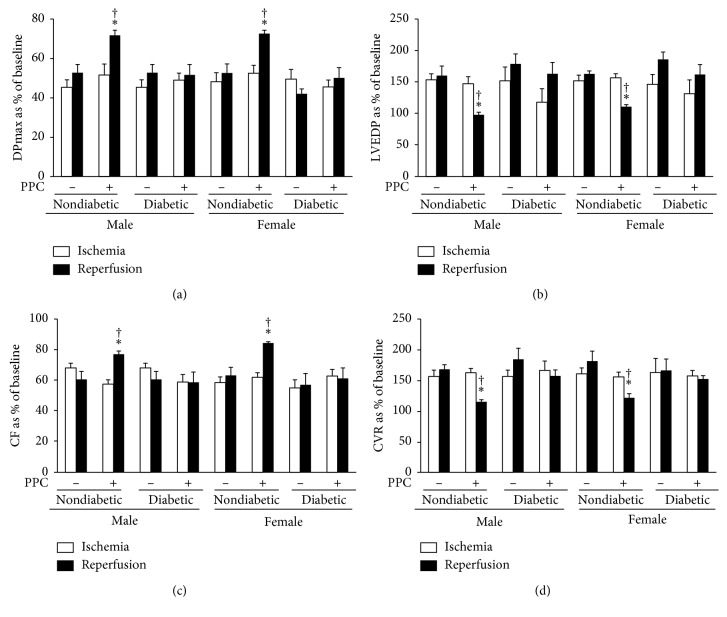
Effects of PPC on left ventricular and coronary vascular dynamics hearts isolated from nondiabetic and diabetic rats. PPC-mediated protection in hearts isolated from diabetic male and female rats (*n*=6 per group). Postischemic recovery from the dysfunction of the heart: (a) DPmax; (b) LVEDP; (c) CF; and (d) CVR. The data were computed at 30 min of reperfusion and are expressed as the means ± SEM. Ctr, control; PPC, pacing postconditioning. ^*∗*^*P* < 0.01 compared to the respective control. ^†^*P* < 0.01 compared to the ischemic period.

Diabetes mellitus did not have a significant effect on cardiac sensitivity to I/R injury (Figures 3 and 5; Tables 1 and 2). PPC did not induce significant improvements in the LV dynamics (Figures 5(a) and 5(b)), LV contractility (Table 1), or coronary vascular dynamics (Figures 5(c) and 5(d)) in hearts isolated from diabetic male and female rats. Furthermore, the presence of diabetes abrogated PPC-induced reduction in infarct size and release of cardiac enzymes following I/R injury equally in male and female rats (Figure 3(c) and Table 2), suggesting that under the conditions tested, PPC is not an efficient cardioprotective method in the diabetic rats.

### 3.4. PPC-Induced Cardiac Protection Is Preserved in Pregnant Rats

**Figure 6:**
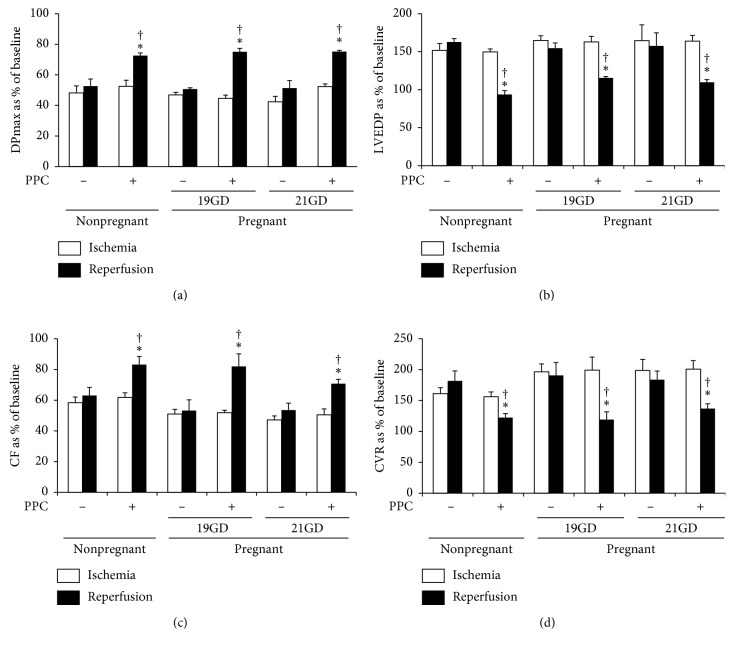
Effects of PPC on left ventricular and coronary vascular dynamics in hearts isolated from nonpregnant and pregnant rats. PPC-mediated protection in hearts isolated from pregnant rats at 19 or 21 gestation days (*n*=6 per group). Postischemic recovery from the dysfunction of the heart: (a) DPmax; (b) LVEDP; (c) CF; and (d) CVR. The data were computed at 30 min of reperfusion and are expressed as the means ± SEM. Ctr, control; PPC, pacing postconditioning; GD, gestation day. ^*∗*^*P* < 0.01 compared to the respective control. ^†^*P* < 0.01 compared to the ischemic period.

To address the effectiveness of PPC in the protection against I/R injury during pregnancy, hearts from nonpregnant and pregnant females at 19 and 21 days of gestation were subjected to I/R injury-followed PPC. The sensitivity of hearts from pregnant rats at 19 and 21 days of gestation to I/R injury was not significantly different from that of hearts isolated from nonpregnant rats. PPC significantly normalized LV dynamics (*P* < 0.01), LV contractility (*P* < 0.001), and vascular dynamics (*P* < 0.01) in nonpregnant and pregnant rats (Figure 6 and Table 1). This effect was further confirmed by significant reductions in the infarct size (*P* < 0.001) (Figure 3(d)) and cardiac enzyme levels (*P* < 0.01; Table 2). These data indicate for the first time that PPC is an effective cardioprotective maneuver against I/R injury in pregnant rats.

## 4. Discussion

**Figure 7:**
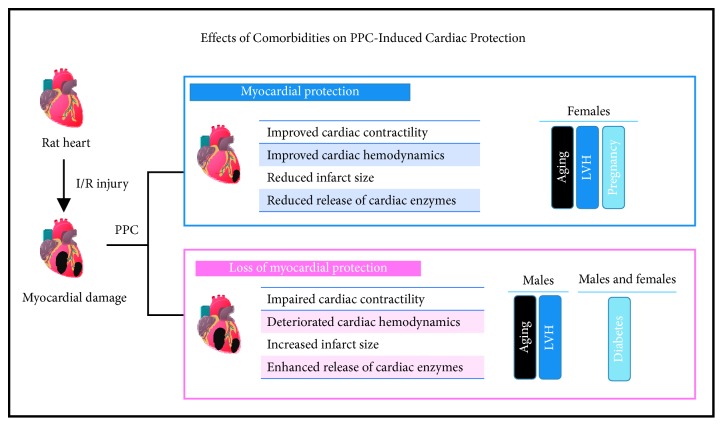
Effects of comorbidities on PPC-induced cardiac protection. Schematic representation summarizing the results of the study.

In the present study, we investigated the sensitivity of hearts isolated from male and female rats with clinically relevant scenarios (aging, LVH, diabetes mellitus, and pregnancy) to ischemic injury and the efficacy of PPC maneuver in protecting these hearts against I/R injury. We demonstrate that aging, LVH, diabetes mellitus, and pregnancy do not affect heart sensitivity to ischemic injury. Furthermore, our data suggest the existence of a novel gender-related discrepancy in PPC-induced cardiac protection in aged and spontaneously hypertensive rats. Heart protection in these animals was exclusively restricted to females. This, however, was not observed in diabetic rats. PPC-induced cardiac protection was revoked equally in diabetic male and female rats. Finally, we demonstrate, for the first time that PPC is an effective cardioprotective maneuver in pregnant rats (Figure 7).

The first clinical application of PPC was conducted by the Waltenberger and Prinzen groups when they demonstrated encouraging results on the potency of PPC in reducing the infarct size in STEMI patients. This, however, was accompanied by atrial and ventricular fibrillation in about 23% of the population, suggesting the requirement of additional technical optimization [11]. We realize that the successful clinical application of PPC maneuver would, therefore, require a detailed, in-depth analysis of various clinical scenarios existing in MI patients.

The protective effects of ischemic postconditioning were attenuated in aged mice [37]. We have therefore tested if PPC remains effective in protecting hearts from aged male and female rats against I/R injury. Our data suggest that aging does not enhance cardiac sensitivity to ischemic injury. Consistent with our previous data in rabbits, PPC was equally protective in younger male and female rats [25]. In aged rats, however, PPC was protective in a gender-specific manner. PPC protected the hearts isolated from 20-month-old female rats but not from male rats against I/R injury. The observed resistance of aged male rats to cardioprotection is consistent with other studies [38, 39]. The exact reason(s) behind gender-related discrimination in cardioprotection is not understood. Several functional alterations were reported in intact hearts and in cardiomyocytes isolated from aged males. Dysfunctional regulation of mitochondrial permeability transition pore (mPTP) opening has been implicated in the loss of the cardioprotective effects of cyclosporine A in aged male cardiomyocytes [38]. Oxidative stress-induced mitochondrial damage was further reported in aged male hearts during ischemia resulting in mitochondria-derived cardiomyocytes injury [40]. Moreover, a significant increase in myocardial protein oxidation was reported in aged male hearts relative to younger controls [41]. Presence of basic lower levels of oxidants and higher levels of antioxidants in the female heart may explain the better protection to the female heart compared to male [42] which is attributed to the different nature of the mitochondrial functioning in male and female hearts [43]. Female hearts were proven to have higher levels of the protective phosphorylated AKT, GSK-3*β*, PKC, and anti-apoptotic factor BCl-2 [42]. Increased levels of the protective phosphorylated AKT, glycogen synthase kinase 3*β* (GSK-3*β*), PKC, and anti-apoptotic factor BCl-2 were reported in hearts from female rodents [42]. Moreover, alterations in cardiac inflammasome with aging could contribute to the observed resistance of aged male hearts to PPC-induced cardiac protection. Enhanced myocardial gene expression of proinflammatory cytokines was reported in animals [44] and clinical studies [45]. Collectively, this could potentially explain a lack of PPC-induced cardiac protection in males. Although protection to female rats could be due to the presence of the female hormone estrogen [6, 46], the exact cardioprotective factors conferring protection to females remain to be identified. A side-by-side comparison between males and females is required to identify possible female-specific cardioprotective factor(s).

Our data suggest that LVH does not increase cardiac sensitivity to ischemic injury. Moreover, we demonstrate that hearts from aged, 20-month-old, spontaneously hypertensive male rats are resistant to PPC-mediated cardioprotection. This was not the case in females where hearts were not hypertrophied and were responsive to PPC-induced cardiac protection as evident by reduction in the infarct size and cardiac enzyme release and normalization of contractility and coronary measures. We have previously demonstrated that estrogen receptor-beta protected murine female hearts from LVH [47]. The resistance of hypertrophied male hearts to PPC-mediated cardiac protection is consistent with other studies investigating the cardioprotective effects of captopril postconditioning [18, 19]; however, contrasting finding was also reported [48]. Attenuated signaling via reperfusion injury salvage kinase (RISK) GSK-3*β* [49] and PI3K [50] were proposed as possible mechanisms of resistance to cardioprotection in hypertrophied hearts.

Here, we report that diabetes mellitus abolishes PPC-induced cardiac protection in a gender-independent manner. The finding that male and female hearts are equally affected by diabetes mellitus possibly suggests that cellular, metabolic, and physiological changes induced by diabetes are so dramatic that cannot be compensated for by factor(s) usually protecting female hearts against I/R injury. Diabetes-induced alterations in intracellular signaling pathways including the PI3K/Akt/GSK-3*β* pathway and enhanced inflammation and oxidative stress were suggested as possible underlying mechanisms for the lack of protection [51–54]. Finally, taking into consideration the pronounced effects of diabetes mellitus on cardiac functions and the signaling pathways of postconditioning protection, the identification of an effective cardioprotective maneuver(s) in the diabetics remains essential.

We demonstrate that pregnancy does not affect the sensitivity of hearts to ischemic injury and PPC stands as an effective maneuver in reducing the infarct size and cardiac enzyme release and normalizing LV contractility and hemodynamics in hearts from pregnant rats. This could be due to pregnancy-induced increase in estrogen levels [55] which was reported to be cardioprotective. Estrogen-mediated cardiac protection involved inhibition of L-type calcium channel, opening of mito-KATP [56–58], and activation of mediators of the RISK pathway [59]. Nevertheless, the exact involvement of estrogen in PPC-induced cardiac protection in pregnant rats' hearts remains to be directly tested.

A potential limitation of this study is that the underlying molecular mechanisms were not investigated. The reason for that is the previous intensive studies conducted on the signaling pathways of postconditioning (RISK and SAFE pathways). These pathways were proven to be involved in the protection of almost all conditioning studies. The lack of heart protection presented in this study could be due to the alteration or remodeling of these signaling pathways by the clinical scenarios which revoked the protection. The signaling pathways of postconditioning protection reported to date in the literature explain the possible inducers for the protection and the lack of protection. We suggest some studies to unravel the alteration and remodeling of signaling pathways caused by comorbidities and other clinical scenarios.

We conclude that IHD in the presence of clinically relevant scenarios including aging, LVH, diabetes mellitus, and pregnancy does not affect cardiac sensitivity to ischemic injury. However, the efficacy of PPC as an effective cardioprotective maneuver is gender-dependent in aged and spontaneously hypertensive rats. PPC protected pregnant rats and loses its cardioprotective properties in diabetic rats in both genders equally.

## Figures and Tables

**Table 1:** Effects of ischemia-reperfusion and PPC on heart contractility.

Treatment	+dP/dt		−dP/dt	
	Ischemia	Reperfusion	Ischemia	Reperfusion
Ctr male	54.32 ± 3.48	58.89 ± 6.94	49.22 ± 2.09	48.28 ± 3.77
Ctr male + PPC	50.66 ± 3.55	80.45 ± 4.64^*∗*^	50.13 ± 3.83	80.22 ± 2.48^*∗*^
Ctr old male	55.30 ± 4.31	56.13 ± 4.53	56.32 ± 4.13	49.53 ± 3.94
Old male + PPC	49.78 ± 1.86	49.66 ± 2.88	58.86 ± 3.81	61.51 ± 4.33
Ctr female	54.59 ± 4.41	48.16 ± 6.70	52.45 ± 3.27	50.81 ± 3.99
Ctr female + PPC	50.66 ± 3.55	75.75 ± 3.43^*∗*^	50.13 ± 3.83	80.22 ± 2.48^*∗*^
Ctr old female	49.22 ± 2.09	48.28 ± 3.77	60.22 ± 4.14	59.91 ± 5.43
Ctr old female + PPC	56.08 ± 3.10	69.08 ± 4.75^*∗*^	56.62 ± 4.65	68.56 ± 2.77^*∗*^
Ctr male	59.31 ± 6.34	64.88 ± 4.99	54.54 ± 4.94	50.74 ± 3.07
Ctr male + PPC	50.66 ± 4.10	81.27 ± 3.02^*∗*^	52.57 ± 2.46	78.81 ± 3.66^*∗*^
Ctr LVH male	66.45 ± 5.47	76.57 ± 4.43	67.44 ± 2.43	76.99 ± 3.53
LVH male + PPC	69.32 ± 6.87	68.64 ± 2.78	68.74 ± 8.50	80.72 ± 5.22
Ctr female	53.82 ± 6.07	55.56 ± 6.25	54.77 ± 3.29	57.84 ± 5.05
Ctr female + PPC	62.63 ± 4.68	82.08 ± 2.96^*∗*^	61.76 ± 4.05	78.49 ± 4.64^*∗*^
Ctr LVH female	68.20 ± 7.11	72.83 ± 6.33	73.27 ± 9.72	71.35 ± 4.48
LVH female + PPC	56.99 ± 7.82	85.57 ± 7.14^*∗*^	52.61 ± 3.51	70.46 ± 6.42^*∗*^
Ctr diabetic male	59.31 ± 6.34	64.88 ± 4.99	54.54 ± 4.94	50.74 ± 3.07
Diabetic male + PPC	55.86 ± 4.02	59.29 ± 8.94	49.06 ± 2.68	47.53 ± 4.77
Ctr diabetic female	58.98 ± 5.09	55.04 ± 8.03	56.15 ± 3.46	58.39 ± 4.79
Diabetic female + PPC	53.05 ± 4.18	59.70 ± 8.90	47.84 ± 2.02	48.16 ± 4.68
Ctr female 19 GD	49.45 ± 2.59	63.63 ± 1.81	51.97 ± 1.24	65.17 ± 0.66
Female 19 GD + PPC	50.46 ± 3.36	72.59 ± 1.38^*∗*^	55.97 ± 1.57	71.27 ± 1.29^*∗*^
Ctr female 21 GD	47.19 ± 2.04	59.78 ± 1.46	47.84 ± 1.69	64.00 ± 0.43
Female 21 GD + PPC	49.79 ± 2.09	72.80 ± 1.88^*∗*^	50.95 ± 3.28	71.19 ± 0.63^*∗*^

Ctr: control; PPC: pacing postconditioning; LVH: left ventricle hypertrophy; GD: gestation day. ^*∗*^*P* < 0.01 compared to the respective control.

**Table 2:** Effects of ischemia-reperfusion and PPC on cardiac enzymes levels.

Treatment	CK IU/L	*P* value	LDH IU/L	*P* value
Ctr male	21.32 ± 1.84	—	20.73 ± 1.37	—
Ctr male + PPC	9.41 ± 1.87^*∗*^	0.001	7.94 ± 1.83^*∗*^	0.001
Ctr old male	21.13 ± 1.12	—	18.62 ± 2.10	—
Old male + PPC	20.23 ± 1.43	0.231	18.42 ± 2.02	0.411
Ctr female	20.13 ± 1.82	—	18.93 ± 1.11	—
Ctr female + PPC	10.21 ± 1.71^*∗*^	0.001	9.95 ± 1.64^*∗*^	0.001
Ctr old female	20.13 ± 1.86	—	16.47 ± 1.82	—
Old female + PPC	10.53 ± 1.24^*∗*^	0.001	9.03 ± 1.65^*∗*^	0.001
Ctr male	23.41 ± 2.13	—	19.21 ± 1.98	—
Ctr male + PPC	10.23 ± 1.43^*∗*^	0.001	8.11 ± 2.13^*∗*^	0.001
Ctr LVH male	23.54 ± 2.63	—	16.22 ± 1.43	—
LVH male + PPC	21.39 ± 2.02	0.315	17.53 ± 1.45	0.314
Ctr female	20.85 ± 1.93	—	17.97 ± 1.36	—
Ctr female + PPC	10.98 ± 1.61^*∗*^	0.001	9.14 ± 1.82^*∗*^	0.001
Ctr LVH female	22.10 ± 1.83	—	16.31 ± 1.92	—
LVH female + PPC	9.14 ± 1.19^*∗*^	0.001	10.63 ± 1.92^*∗*^	0.01
Ctr diabetic male	23.75 ± 2.13	—	17.52 ± 1.61	—
Diabetic male + PPC	21.72 ± 1.71	0.524	19.27 ± 2.13	0.243
Ctr diabetic female	20.63 ± 2.85	—	18.92 ± 1.62	—
Diabetic female + PPC	21.31 ± 1.86	0.463	18.26 ± 1.23	0.217
Ctr female 19 GD	20.42 ± 1.66	—	16.24 ± 1.23	—
Female 19 GD + PPC	9.92 ± 1.12^*∗*^	0.001	8.31 ± 1.14^*∗*^	0.001
Ctr female 21 GD	21.33 ± 1.62	—	17.24 ± 1.38	—
Female 21 GD + PPC	9.87 ± 1.95^*∗*^	0.001	8.05 ± 1.23^*∗*^	0.001

CK: creatine kinase; LDH: lactate dehydrogenase; Ctr: control; PPC: pacing postconditioning; LVH: left ventricle hypertrophy; GD: gestation day. ^*∗*^*P* < 0.001 compared to the respective control.

## Data Availability

The data used to support the findings of this study are included within the article.

## Abbreviations

LVH: Left ventricle hypertrophy
IHD: Ischemic heart disease
PPC: Pacing postconditioning
I/R: Ischemia and reperfusion
MI: Myocardial infarction
CVDs: Cardiovascular diseases
SD: Sprague–Dawley
SHR: Spontaneously hypertensive rat
STZ: Streptozotocin
LVEDP: Left ventricle end-diastolic pressure
DPmax: Maximum developed pressure
CF: Coronary flow
CVR: Coronary vascular resistance
TTC: Triphenyltetrazolium chloride control
CK: Creatine kinase
LDH: Lactate dehydrogenase
Ctr: Control
GD: Gestation day.
